# Thymic Self-Antigen Expression for the Design of a Negative/Tolerogenic Self-Vaccine against Type 1 Diabetes

**DOI:** 10.1155/2011/349368

**Published:** 2011-05-23

**Authors:** Aziz Alami Chentoufi, Vincent Geenen

**Affiliations:** ^1^Department of Immunology, Faculty of Medicine, King Fahad Medical City, Riyadh, Saudi Arabia; ^2^University of Liege Center of Immunology, Institute of Pathology CHU-B23, B-4000 Liege-Sart Tilman, Belgium

## Abstract

Before being able to react against infectious non-self-antigens, the immune system has to be educated in the recognition and tolerance of neuroendocrine proteins, and this critical process essentially takes place in the thymus. The development of the autoimmune diabetogenic response results from a thymus dysfunction in programming central self-tolerance to pancreatic insulin-secreting islet **β** cells, leading to the breakdown of immune homeostasis with an enrichment of islet **β** cell reactive effector T cells and a deficiency of **β** cell-specific natural regulatory T cells (nTreg) in the peripheral T-lymphocyte repertoire. Insulin-like growth factor 2 (IGF-2) is the dominant member of the insulin family expressed during fetal life by the thymic epithelium under the control of the autoimmune regulator (*AIRE*) gene/protein. Based on the close homology and cross-tolerance between insulin, the primary T1D autoantigen, and IGF-2, the dominant self-antigen of the insulin family, a novel type of vaccination, so-called “negative/tolerogenic self-vaccination”, is currently developed for prevention and cure of T1D. If this approach were found to be effective for reprogramming immunological tolerance in T1D, it could pave the way for the design of negative self-vaccines against autoimmune endocrine diseases, as well as other organ-specific autoimmune diseases.

## 1. Introduction

Type 1 diabetes (T1D) is a chronic autoimmune disorder associated, in genetically susceptible individuals, with the generation and activation of autoreactive T cells recognizing pancreatic *β* cell autoantigens. Autoreactive CD4+ and CD8+ T cells infiltrate pancreatic Langerhans' islets (insulitis) and selectively destroy the insulin-producing *β* cells in these structures. This destruction occurs silently and progressively and, in most cases, remains undetected for many years. By the time the first clinical T1D symptoms become apparent, nearly 80% of the patients' *β* cells have been destroyed and there is little hope for curing the disease. In humans, T1D incidence peaks around 10–14 years of age. It is estimated that T1D affects about 30 million people worldwide (approximately 10–15% of all patients with diabetes mellitus). 

Major T1D autoantigens include (pro)insulin, the 65-kDa isoform of glutamic acid decarboxylase (GAD65), the islet tyrosine phosphatase IA-2, and the islet-specific glucose-6-phosphatase catalytic subunit-related protein (IGRP). These pancreatic *β*-target autoantigens have been identified in both humans and nonobese diabetic (NOD) mice [1]. The generation of autoreactivity to islet *β* cells is the consequence of multiple various genetic defects that have an impact on fundamental immunological processes such as central and peripheral tolerance. In general, potentially autoreactive lymphocytes must encounter their cognate autoantigen during differentiation in order for the host to develop tolerance to self-antigens. This encounter may occur within the thymus or upon release into the periphery [2, 3]. Central self-tolerance occurs during T-cell differentiation in the thymus and involves deletion (negative selection) of self-reactive T cells during T-cell development in the thymus [4]. This process occurs to eliminate T cells that recognize ubiquitous or blood-borne self-antigens. T cells which recognize tissue-specific antigens (TSAs), however, were long-thought to be tolerized in the periphery [3]. Until recently, it was widely assumed that such TSAs are excluded from the thymus, precluding central self-tolerance. To explain tolerance to TSAs, numerous peripheral tolerance mechanisms have been suggested, including ignorance and immunoregulation. At the same time it has also been argued that TSAs may reach the thymus in significant quantities and thereby induce negative selection *in situ*, especially of high-affinity T cells. This idea became more plausible when others and we found that many TSAs are expressed by rare medullary thymic epithelial cells (mTEC) [5–7]. Findings such as these challenged historical assumptions about tolerance to TSAs and intense investigations, in both mice and humans, have elucidated the identity of TSA-expressing cells in the thymus, as well as their role in the process of central self-tolerance. Others and we showed that genetically determined low levels of TSAs (i.e., insulin) expression in the thymus is associated with the generation of high numbers of autoreactive T cells in the periphery and predisposition to autoimmune disease [5–7]. The ectopic expression of many tissue-restricted antigens in the thymus is controlled by the autoimmune regulator (*AIRE*). *AIRE* is a 54.5-kDa protein with a nuclear localization and several potential DNA-binding and protein interaction domains [8]. *AIRE* ^−/−^ mice exhibit a defined profile of autoimmune diseases including T1D. It is notable that all these diseases arise due to a lack of *AIRE* expression in stromal cells of the thymus. Furthermore, *AIRE*-deficient mTEC showed a specific reduction in promiscuous transcription of genes encoding peripheral antigens, demonstrating the importance of thymus-dependent tolerance in controlling autoimmunity [9].

Although these data are appealing, they do not prove that thymic expression of TSAs automatically induces central self-tolerance to peripheral tissues. Other potential mechanisms for developing tolerance to TSAs also include thymic generation of self-antigen-specific Foxp3+CD4+CD25+ regulatory T cells or Treg (“dominant” central self-tolerance). In the last 25 years, Foxp3+CD4+CD25+ Treg have been identified as key effectors in the maintenance of peripheral tolerance. It has also been shown that the promiscuous expression of a neo-self-antigen in TEC may be involved in the selective induction and/or expansion of self-antigen-specific Foxp3+CD4+CD25+ Treg thymic precursors as early as the double-positive stage [10]. Importantly, this selection of self-antigen-specific Foxp3+ Treg is mediated by *AIRE*+ mTEC [11]. In this paper, we highlight the recent progress in the field of central and peripheral self-tolerance exemplified by T1D pathogenesis as consequence of a deleterious process in T-cell tolerance installment.

## 2. T1D-Associated Autoantigens and Autoimmunity

An important step to demonstrate the autoimmune nature of T1D was the discovery of autoantibodies directed against Langerhans' islet cells [12]. Major autoantigens include (pro)insulin itself, GAD65 and IGRP. The islet-specific cation efflux transporter ZnT8 (Slc30A8) and chromogranin A have been also recently reported as important autoantigens in T1D [13, 14]. However, among these autoantigens, only antigenic epitopes derived from (pro)insulin are specific of pancreatic islet *β* cells. Furthermore, there is now ample evidence that autoimmunity to (pro)insulin is central to autoimmune diabetes pathogenesis both in non-obese diabetic (NOD) mice and in humans [15, 16]. Clinically, detection of anti-insulin, anti-GAD65, and/or anti-IA-2 autoantibodies are very reliable markers of the autoimmune response targeting *β* cells. The majority of the children with recent-onset of T1D (more than 90%) have antibodies against one or several of these autoantigens in their serum. Their high predictive value is also established since they can be detected several years before the clinical signs of insulin deficiency. The predictive value of autoantibodies against several autoantigens is higher than elevated titers of one single autoantibody. The combination of autoantibodies with susceptible genetic alleles of the major histocompatibility (MHC) class II locus further increases this predictive value. Such prediction is very useful for clinical studies targeted at T1D prevention given the relatively low incidence of this disease [17, 18]. However, the pathogenic significance of T1D-related autoantibodies is rather low, if not absent [19], and the principal effectors of *β* cell autoimmune destruction are CD4+ and CD8+ T lymphocytes [20]. Investigation of specific T-cell responses in T1D patients is however challenging because of the very low frequency in the peripheral T-cell pool of autoreactive T cells specific for epitopes derived from (pro)insulin, GAD65, or IA-2. However, the development of sensitive and specific techniques, such as enzyme-linked immunosorbant spot assays (ELISpot) and multimers of class I/II HLA molecules complexed with T1D-related epitopes, has already provided very significant data that further document the importance of T-cell-mediated mechanisms in T1D pathogenesis [21, 22].

## 3. The Central Role of the Thymus in Central Self-Tolerance of Neuroendocrine Proteins and the Nature of “Neuroendocrine Self”

A major question when addressing the pathogenesis of organ-specific autoimmunity such as T1D is the origin of the self-reactive T cells that are directed against target antigens of endocrine cells. Among all lymphoid structures, the thymus is an organ that emerged some 500 million years ago, concomitantly or very shortly after recombinase-dependent adaptive immunity, with a specific function in orchestrating central immunological self-tolerance. The thymus is not an endocrine gland, but it crucially stands at the intersection between the immune and neuroendocrine systems. In this organ that is responsible for thymopoiesis, that is, generation of naïve and competent T lymphocytes, the neuroendocrine system regulates the process of T-cell differentiation from very early stages, while in parallel naïve T lymphocytes are educated to recognize and tolerate neuroendocrine gene/protein families [7, 23, 24]. Contrary to the popular opinion, the thymus continues to function throughout life and plays a fundamental role in the recovery of a competent T-cell repertoire after intensive chemotherapy or during highly active antiretroviral chemotherapy in human immunodeficiency virus infection [25, 26]. The integrity of the somatotrope growth hormone/IGF-1 axis is known to be important for the maintenance of thymus function in adult life [27]. 

The thymus is the central lymphoid organ responsible for the maturation and differentiation of bone-marrow-derived thymocytes. The random generation of the T-cell repertoire, including autoreactive T cells, is regulated in the thymus by mechanisms of central self-tolerance. Anatomically, the thymus is divided into subcapsular, cortical, and medullary compartments. The stromal cells include a variety of bone-marrow-derived professional antigen-presenting cells (dendritic cells [DC], macrophages, and B cells) and endoderm-derived cortical (c) TEC and mTEC [28]. A striking morphologic feature of the medulla is the presence of Hassall's corpuscles, which consist of concentric whorls of stratified keratinizing epithelium and share antigenic properties with ectodermic epithelium [29]. The thymus constitutes the central arm of immunological self-tolerance by two essential mechanisms that are intimately associated and paradoxically mediated by the same thymic self-antigens: (a) negative selection of self-reactive T cells issued from the random recombination of TCR genes (“recessive” self-tolerance) and (b) generation of self-antigen-specific nTreg that are able to inactivate in periphery self-reactive T cells having escaped intrathymic negative selection (“dominant” self-tolerance) [30, 31].

Several groups and ourselves have demonstrated that TEC from different species constitute a site for the promiscuous transcription of a great number of genes encoding tissue-restricted antigens or belonging to neuroendocrine families, such as the neurohypophysial family, tachykinins, neurotensins, somatostatins, atrial natriuretic peptides, and the insulin family. This demonstration has radically changed our common understanding of the pathogenesis of organ-specific autoimmune endocrine diseases such as T1D. From the investigation of intrathymic expression of neuroendocrine-related self-peptide precursor genes, a series of properties can be derived that define the nature of the “neuroendocrine self”. First, thymic neuroendocrine self-antigens usually correspond to peptide sequences that have been highly conserved throughout the evolution of their related family. Second, a hierarchy characterizes their expression pattern in the thymus. In the neurohypophysial family, oxytocin (OT) is the dominant peptide synthesized by TEC from different species. The binding of OT to its cognate receptor expressed by pre-T cells induces a very rapid phosphorylation of focal adhesion-related kinases. This event could play a major role in promoting establishment of synapses between immature T lymphocytes and TEC, as well as with macrophages and DC. All the genes of the insulin family are expressed in the thymus according to a precise hierarchy during fetal life: *IGF2* (cTEC and mTEC) >*IGF1*>*INS* (a few subsets of mTEC). This hierarchical pattern is meaningful because the strength of self-tolerance to a protein is proportional to its intrathymic concentration [32]. Third, neuroendocrine precursors are not processed according to the classic model of neurosecretion, but they undergo an antigenic processing for presentation by—or in association with—MHC proteins [33]. Fourth, most of neuroendocrine self-antigens are transcribed in the thymic epithelium under the control of the autoimmune regulator gene *AIRE* (see below). Fifth, intrathymic *OT* transcription precedes OT and vasopressin (VP) expression in hypothalamic magnocellular neurons. Finally, epigenetic regulation of intrathymic gene expression is strongly suggested by the loss of *IGF2* parental imprinting in human mTEC [34, 35].

This hierarchy in the organization of the thymic repertoire of neuroendocrine self-antigens is also significant from an evolutionary point of view. Since a series of essential and physiological functions had been established before the appearance of adaptive immunity in cartilaginous fishes, they had to be protected from the risk of autotoxicity inherent to this type of immunity. For example, OT is a “bonding” peptide that has been implicated at different steps of the reproductive process and thus, for species preservation, OT possibly had to be protected to a greater degree than VP, which controls water metabolism and vascular tone. Along the same line of reasoning, IGF-2 as a major factor in fetal development possibly had to be more protected than insulin, which “only” regulates glucose homeostasis. Nevertheless, because of their close homology, thymic neuroendocrine self-antigens may promote cross-tolerance to other members of their respective families. This is supported by the weaker tolerance to insulin of *Igf2* ^−/−^ mice when compared to wild-type mice [36]. Further insight into the relative influence of the central arm of immunological self-tolerance will be gained through the generation of mice with TEC-specific *Igf2* deletion, currently under development in our laboratory.

## 4. The Central Role of a Thymus Dysfunction in T1D Pathogenesis (Figure 1)

As hypothesized by Burnet in 1973, the pathogenesis of autoimmune diseases may first depend on the appearance of “forbidden” self-reactive clones in the peripheral T-cell repertoire [37]. In 1992, a defect in the process of intrathymic T-cell education to recognize and to tolerate OT was hypothesized to play a pivotal role in the development of hypothalamus-specific autoimmunity leading to “idiopathic” central diabetes insipidus [38]. The progressive increase in the degree of immune diversity and complexity may explain why failures in self-tolerance are increasingly detected during evolution with most such failures occurring in the human species. Since the thymus is the primary site for induction of self-tolerance, a thorough investigation of the mechanisms responsible for a breakdown of thymus-dependent tolerance should provide the scientific community with important keys to understand the mechanisms underlying the development of autoimmune responses. 

A number of abnormalities of thymic morphology and cytoarchitecture have been described in T1D. Central tolerance and apoptosis of self-reactive T cells are defective in the thymus of NOD mouse [39, 40]. Transcription of insulin-related genes (*Ins, Igf1*, and *Igf2*) has been analyzed in the thymus of diabetes-resistant (BBDR) and diabetes-prone (BBDP) rats, another model of T1D. *Ins* and *Igf1* transcripts were detected in all thymi from BBDP and BBDR rats. *Igf2* transcripts were also present in the thymus from all BBDR rats, but were not detected in the thymus from more than 80% of BBDP rats, in close concordance with the incidence (86%) of autoimmune diabetes in those rats. This defect in *Igf2* transcription in BBDP thymus could also explain both their lymphopenia (including CD8+ T cells and RT6+ Treg) and the absence of central self-tolerance to insulin-secreting islet *β* cells [41, 42]. As already mentioned, we have shown that susceptibility to autoimmune diabetes is correlated with the level of *Ins2* transcription in the mouse thymus [6]. Breeding of *Ins2* ^−/−^ mice onto the NOD background markedly accelerated insulitis and onset of diabetes [43]. In contrast, insulitis and diabetes were considerably reduced in *Ins1* ^−/−^ congenic NOD mice [44]. These observations are explained by the dominance of *Ins2* encoding proinsulin in the murine thymus, while *Ins1* encodes proinsulin in islet *β* cells. In the human species, *INS* transcripts were measured at lower levels in the fetal thymus with short class I VNTR (variable number of tandem repeats) alleles, a genetic trait of T1D susceptibility as discussed above [45, 46]. The contribution of thymic insulin in mediating central self-tolerance to islet *β* cells was definitively demonstrated by the rapid onset of autoimmune diabetes following thymus-specific deletion of *Ins1* and *Ins2* through an elegant transgenic construction in mice [47].

The identification of *AIRE* led to further demonstration that a thymus dysfunction plays a crucial role in the pathogenesis of organ-specific autoimmune diseases [48, 49]. Loss-of-function *AIRE* single mutations are responsible for a very rare autosomal recessive disease named autoimmune polyendocrinopathy, candidiasis, and ectodermal dystrophy (APECED), or autoimmune polyendocrine syndrome type 1 (APS-1). This syndrome develops in early childhood and is characterized by multiorgan autoimmunity and insufficiency of several endocrine glands such as parathyroids, adrenal cortex, and gonads. *AIRE* expression is maximal in the thymus, mainly in mTEC, but is absent in TEC of NOD mice [50]. Depending on their genetic background, *AIRE* ^−/−^ mice exhibit several signs of peripheral autoimmunity, which are associated with a significant decrease in thymic transcription of neuroendocrine genes (including *Ot, Npy, Igf2,* and *Ins2*), as well as other TSAs [9, 51, 52]. However, as shown for GAD67, AIRE does not control the intrathymic expression of all TSAs. By different aspects, *AIRE*-regulated transcription in the thymus differs from expression of these antigens in eutopic tissues. For example, loss of *Igf2* imprinting with biallelic transcription has been observed in mTEC [53]. The same study has shown that many TSAs in mTEC are clustered in their chromosomal location, including *AIRE*-dependent and *AIRE*-independent gene targets [53]. Extrathymic *AIRE* expression has been evidenced in secondary lymphoid organs where *AIRE* also controls the expression of TSA genes that are different from those regulated by *AIRE* in mTEC [54]. The precise molecular mechanisms by which *AIRE* controls transcription of TSA are not completely elucidated [55, 56]. However, it more and more appears that *AIRE* uses one of its two zinc finger plant homeodomains for binding to nonmethylated histone H3K4 and activating gene expression, which establishes a relationship between chromatin regulation and tissue-specific central tolerance [57, 58]. Finally, there is mounting evidence that *AIRE* is closely implicated in mTEC differentiation (reviewed in [59]).

In collaboration with Didier Hober (Laboratory of Virology EA3610, CHRU Lille, France), we have shown that coxsackievirus B4 (CVB4) is capable to directly infect the epithelial and lymphoid compartments of the human and murine thymus and to induce a severe thymus dysfunction with massive pre-T-cell depletion and marked upregulation of MHC class I expression by TEC and by CD4+ CD8+ immature thymic T cells [60, 61]. Interestingly, outbred mice can be infected with CVB4 following an oral inoculation, which results in systemic spreading of viral RNA and a prolonged detection of CVB4 RNA in thymus, spleen, and blood up to 70 days postinoculation [62]. These findings suggest that, in addition to a role for CVB4 in breaking peripheral tolerance to islet *β* cells, the severe infection of the thymus by CVB4 could enhance its virulence through induction of central tolerance to the virus, and a still putative breakdown in central self-tolerance to islet *β* cells.

## 5. Thymic Control of Naturally Occurring CD4+CD25+ Treg Generation in T1D

Naturally occurring CD4+CD25+ Treg have emerged as a dominant T-cell population mediating peripheral tolerance. They are potent suppressors of organ-specific autoimmunity (T1D, inflammatory bowel disease, and gastritis), allograft rejection, graft versus host disease and control immunity to asthma and infectious agents such as parasites and viruses [63–68]. Depletion of CD4+CD25+ Treg cells leads to the development of various autoimmune diseases in genetically susceptible animals. In contrast, the adoptive transfer of CD4+CD25+ Treg in many animal models has been shown efficient in controlling organ-specific autoimmunity [69].

Multiple reports have implicated Treg in T1D prevention. Treg depletion or interference with B7/CD28 pathway in NOD mice has been shown to accelerate T1D onset [70, 71]. In aged NOD mice, autoimmune diabetes resistance has been correlated with the expansion of CD25+ CD4+ T cells with regulatory activity within inflamed pancreatic lymph nodes [72]. Recently, Chen et al. reported that Foxp3-deficient NOD mice, which are deficient in Treg, display an increased incidence and earlier onset of T1D compared with normal NOD mice, strongly implying a role for Treg in the control of T1D pathogenesis [73]. Obviously, alterations in the frequency and/or function of Treg are associated with various autoimmune diseases, including T1D [74, 75]. The quantification of naturally occurring Treg is hampered by a lack of unique surface markers. Perhaps not surprisingly, there is some controversy in the literature regarding the frequency of Treg in NOD mice, as determined by the identification of CD4+CD25 high cells in the spleen and lymphoid organs. More importantly, however, it appears that NOD mice harbor Treg that prevent disease development at an early age, but lose their functional capacity later on in life, thereby allowing pathogenic effector T cells to attack pancreatic islets [76, 77]. Similarly, the numbers of Foxp3+ Treg in the peripheral blood of T1D patients do not appear to be significantly different from healthy control subjects but there is evidence for functional defects in their suppressive capacity [78–80]. Therefore, there has been much interest in the potential use of adoptive transfer of *in vitro* expanded islet-specific Treg as a way of suppressing autoimmunity in T1D patients, perhaps preserving islet-cell function in newly diagnosed T1D.

The role of thymic self-antigens expression in the negative selection of self-reactive T cells has been well described by others and us. Many reports have shown that thymic mTEC and subsets of DC also play a critical role in the generation of antigen-specific CD4+CD25+Foxp3+ Treg [10]. Moreover, mTEC forming Hassall's corpuscles in the human thymus express thymic stromal lymphopoietin (TSLP), a molecule that can instruct thymic and peripheral DC to drive thymocyte CD4+CD8−CD25− differentiation into naturally occurring regulatory CD4+CD25+ T-cells [81, 82]. Importantly, Aschenbrenner et al. have shown that this selection of Foxp3+ Treg specific for self-antigen is mediated by *AIRE*+ mTEC, and the “routing” of mTEC-derived self-antigens (i.e., direct presentation by mTEC or via transfer to DC) may determine whether specific thymocytes are deleted or enter the Treg lineage. Indeed, this study proposed that mTEC-thymocyte interactions shape the Treg compartment and dominant tolerance, whereas hematopoietic antigen-presenting cells (mainly thymic DC) mediate recessive deletional tolerance [11]. Taken together, these results show that mTEC forming Hassall's corpuscles play an important role in central and peripheral T-cell tolerance.

## 6. Genetic Factors in T1D Pathogenesis

T1D is the polygenic autoimmune disease that has been most intensively investigated at the genetic level. Knowledge of genetic loci that determine susceptibility to T1D is important for identifying pathogenic pathways, for improved prediction of the disease and for selection of potential pharmacological targets. The balance between susceptibility and resistance alleles determines individual predisposition to T1D. The most significant part (±50%) of genetic susceptibility to T1D resides in the HLA class II region on chromosome 6p21, as recognized by pioneering studies [83, 84]. The major susceptibility in this region is conferred by the specific HLA class II haplotypes DR4-DQA1*0301-DQB1*0302 (DQ8 molecule) and DR3-DQA1*0501-DQB1*0201 (DQ2 molecule). In contrast, the allele DQB1*0602 (DQ6 molecule) confers dominant protection against T1D. Theoretically, HLA class I proteins present antigens that are processed from endogenous proteins to CD8+ T cells, while HLA class II proteins present antigens issued from exogenous proteins to CD4+ T cells. Consequently, it has long been difficult to explain the relationship between insulin and T1D genetic susceptibility located in the HLA class II region. This problem was solved when very elegant crystallographic studies showed that a dominant insulin epitope (InsB9-23) is presented in the binding pocket of DQ8 and DQ2 proteins [85]. Since then, a comprehensive scan of the whole HLA region, combined with potent statistical methods, has linked T1D susceptibility to HLA class I genes *HLA-B* and *HLA-A* [86].

Other genetic linkage and association studies have identified a second locus for T1D susceptibility that corresponds to a high polymorphic minisatellite constituted by a variable number of tandem repeats (VNTR) [87, 88]. This VNTR is embedded on chromosome 11p15 and controls the transcription of the insulin (*INS*) and insulin-like growth factor 2 (*IGF2*) genes downstream. Short VNTR class I alleles contain 20–63 repeats of 14-15 base pairs, while intermediate class II and long class III alleles include 64–139 and 140–210 repeats, respectively. VNTR class I alleles are associated with TID susceptibility, whereas class III alleles confer protection.

The *CTLA4* gene region on chromosome 2q33 is also associated with susceptibility to T1D [89]. The signaling between B7, expressed by professional antigen-presenting cells such as DC and cytotoxic T-lymphocyte-associated protein 4 (CTLA-4), expressed by T cells, plays a pivotal role in peripheral T-cell tolerance. CTLA-4 is expressed neither by thymocytes nor resting T cells, but it is detectable after antigen-mediated T-cell activation and downregulates responses of activated T cells. *Ctla4* deletion in mice results in an extremely severe lymphoproliferative and an autoimmune phenotype with lethal multiorgan tissue destruction [90].

Another mutation in a non-HLA gene conferring significant susceptibility to T1D is a variant of the lymphoid tyrosine phosphatase Lyp gene (*PTPN22*), a suppressor of T-cell activation [91]. Lyp normally interacts with a C-terminal Src kinase (Csk) complex to dephosphorylate positive regulatory tyrosines and downregulate signaling from the TCR pathway. The minor allele derived from the single nucleotide polymorphism (SNP) differs in a single but crucial amino acid residue (R620W) involved in interaction of Lyp with Csk. Interestingly, the same variant R620W also increases risk to other common autoimmune diseases, such as rheumatoid arthritis, Graves' disease, and systemic lupus erythematosus [92]. However, the variant *PTPN22* 620W is a gain-of-function mutant, since it is associated with a higher catalytic activity of the encoded Lyp, a marked decrease of T-cell response to antigen stimulation, CD25 expression and IL-10 secretion from TCR stimulation, and an increase in peripheral memory CD4+ T cells [93, 94]. The role of this mutation in the pathogenesis of T1D and other autoimmune diseases remains to be further elucidated.

Different studies, including a genome-wide association analysis, have identified association of T1D with noncoding SNPs on the chromosome 10p15 region containing CD25, which encodes the high-affinity *α* chain of the IL2R complex [95]. Further mapping of the association between the IL2RA locus and T1D supported a role of IL2R*α* in the pathogenesis of the disease, most possibly through modulation of Treg activity [96].

An association has also been found between T1D and a polymorphism of the *IGF2* receptor gene (*IGF2R*), which seems to be subject to parental imprinting since only maternal alleles at this polymorphism are associated with the disease [97]. Human T1D differs from other common autoimmune disorders, which preferentially affect females (like autoimmune diabetes in the NOD mouse). Evidence was also recently provided for an association between T1D and polymorphisms in *CYP27B1*, which encodes 1*α*-hydroxylase, the enzyme that transforms 25(OH) vitamin D into bioactive 1,25(OH)_2_ vitamin D3 [98].

The association between T1D and viral infections has been recently reinforced by genetic studies that have evidenced a linkage between T1D susceptibility and host genetic determinants of the antiviral responses such as the antiviral oligoadenylate synthetase (*OAS1*) and the interferon-induced helicase (*IFIH1* or *MDA5*), which intervenes in innate immunity by recognition of RNA genomes of picornaviruses (such as coxsackie viruses) [99–101]. Therefore, the question of a higher incidence of enterovirus infection during childhood in countries with a high-risk of T1D deserves to be further investigated, particularly if one seriously considers the possibility of anticoxsackie virus vaccination as a potential method for T1D prevention.

## 7. Prospective: The Concept of Negative/Tolerogenic Self-Vaccination

Because of its antigen-specificity, the most attractive immunomodulating approach against the development of the diabetogenic autoimmune response is the design of peptide-based therapeutic vaccines. According to the novel knowledge gained in T1D pathogenesis and the central role of a thymus dysfunction in its development, the control of the autoimmune process could be obtained by reprogramming *β* cell tolerance through the potent tolerogenic properties of the thymus, in particular the repertoire of thymic T1D-related self-antigens. Contrary to insulin, the “altered self-IGF-2,” IGF-2, and derived epitopes might be an appropriate choice for a novel type of a negative self-vaccination that associates competition for MHC presentation and regulatory responses downstream, as well as potential bystander suppression of autoimmune responses to other T1D-related autoantigens. This hypothesis is currently being investigated by vaccination of NOD mice with recombinant human IGF-2 alone or in combination with tolerogenic adjuvants.

## 8. Conclusion

The thymus plays a central role in the establishment of central immunological self-tolerance towards Langerhans' insulin-secreting islet *β* cells, and there is now evidence that T1D development results from a breakdown of thymus-dependent tolerance to insulin family-derived epitopes. This knowledge should translate in the very near future to the design of novel tolerogenic/regulatory approaches aimed at restoring the immunological tolerance specific of islet *β* cells, which represents an appealing strategy for both prevention and cure of T1D, one of the heaviest prices paid by the human species for having evolved the advantage of the extreme diversity and efficiency of adaptive immune responses against new biological threats.

## Figures and Tables

**Figure 1:**
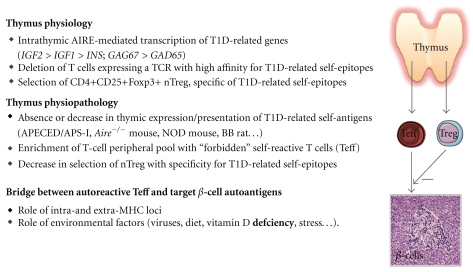
Thymus physiopathology and T1D development. Throughout life, the thymus selects self-tolerant and competent T cells against non-self-antigens and generates self-specific nTreg. Under control by *AIRE* for most of them, thymic epithelium transcribes genes encoding T1D-related antigens, as well as other neuroendocrine-related and tissue-restricted antigens. Absence or decrease in presentation of thymic T1D-related antigens (as observed in different animal models of autoimmune diabetes) conducts to the enrichment of the peripheral T-cell pool with “forbidden” self-reactive T cells (Teff) bearing a TCR directed against T1D-related epitopes, while thymic generation of specific nTreg is severely impaired. Combination of these two events is responsible for the breakdown of central self-tolerance to islet *β* cells. Both genetic and environmental factors are involved in the establishment of a molecular bridge between anti-*β* cell self-reactive Teff and islet *β* cell autoantigens. Once this bridge is formed, the autoimmune pathogenic response is triggered and leads to a progressive destruction of the *β* cell mass.
